# A Deeper Insight into Evolutionary Patterns and Phylogenetic History of ASFV Epidemics in Sardinia (Italy) through Extensive Genomic Sequencing

**DOI:** 10.3390/v13101994

**Published:** 2021-10-04

**Authors:** Mariangela Stefania Fiori, Daria Sanna, Fabio Scarpa, Matteo Floris, Antonello Di Nardo, Luca Ferretti, Federica Loi, Stefano Cappai, Anna Maria Sechi, Pier Paolo Angioi, Susanna Zinellu, Roberto Sirica, Eloisa Evangelista, Marco Casu, Giulia Franzoni, Annalisa Oggiano, Silvia Dei Giudici

**Affiliations:** 1 Department of Animal Health, Istituto Zooprofilattico Sperimentale della Sardegna, 07100 Sassari, Italy; mariangela.fiori@izs-sardegna.it (M.S.F.); annamaria.sechi@izs-sardegna.it (A.M.S.); pierpaolo.angioi@izs-sardegna.it (P.P.A.); susanna.zinellu@izs-sardegna.it (S.Z.); giulia.franzoni@izs-sardegna.it (G.F.); annalisa.oggiano@izs-sardegna.it (A.O.); silvia.deigiudici@izs-sardegna.it (S.D.G.); 2 Department of Biomedical Sciences, University of Sassari, 07100 Sassari, Italy; darsanna@uniss.it (D.S.); matteo.floris@gmail.com (M.F.); 3 Department of Veterinary Medicine, University of Sassari, 07100 Sassari, Italy; fscarpa@uniss.it (F.S.); marcasu@uniss.it (M.C.); 4 The Pirbright Institute, Ash Road, Pirbright, Woking GU24 0NF, UK; antonello.di-nardo@pirbright.ac.uk; 5 Big Data Institute, Nuffield Department of Medicine, University of Oxford, Oxford OX1 4BH, UK; luca.ferretti@gmail.com; 6 Osservatorio Epidemiologico Veterinario Regionale, Istituto Zooprofilattico Sperimentale della Sardegna, 09125 Cagliari, Italy; stefano.cappai@izs-sardegna.it; 7 Ames Polydiagnostic Group Center SRL, 80013 Napoli, Italy; roberto.sirica@centroames.it (R.S.); elo.evangelista@gmail.com (E.E.)

**Keywords:** african swine fever virus, next-generation sequencing, molecular dating, whole genome, phylogeny, molecular epidemiology

## Abstract

African swine fever virus (ASFV) is the etiological agent of the devastating disease African swine fever (ASF), for which there is currently no licensed vaccine or treatment available. ASF is defined as one of the most serious animal diseases identified to date, due to its global spread in regions of Africa, Europe and Asia, causing massive economic losses. On the Italian island of Sardinia, the disease has been endemic since 1978, although the last control measures put in place achieved a significant reduction in ASF, and the virus has been absent from circulation since April 2019. Like many large DNA viruses, ASFV mutates at a relatively slow rate. However, the limited availability of whole-genome sequences from spatial-localized outbreaks makes it difficult to explore the small-scale genetic structure of these ASFV outbreaks. It is also unclear if the genetic variability within outbreaks can be captured in a handful of sequences, or if larger sequencing efforts can improve phylogenetic reconstruction and evolutionary or epidemiological inference. The aim of this study was to investigate the phylogenetic patterns of ASFV outbreaks between 1978 and 2018 in Sardinia, in order to characterize the epidemiological dynamics of the viral strains circulating in this Mediterranean island. To reach this goal, 58 new whole genomes of ASFV isolates were obtained, which represents the largest ASFV whole-genome sequencing effort to date. We provided a complete description of the genomic diversity of ASFV in terms of nucleotide mutations and small and large indels among the isolates collected during the outbreaks. The new sequences capture more than twice the genomic and phylogenetic diversity of all the previously published Sardinian sequences. The extra genomic diversity increases the resolution of the phylogenetic reconstruction, enabling us to dissect, for the first time, the genetic substructure of the outbreak. We found multiple ASFV subclusters within the phylogeny of the Sardinian epidemic, some of which coexisted in space and time.

## 1. Introduction

African swine fever (ASF) is a complex and highly lethal hemorrhagic disease that affects domestic pigs and different species of wild swine. ASF has been defined by the World Organization for Animal Health (OIE) as one of the most dangerous and persistent animal diseases [1]. Historically present in Africa and Europe, the disease has recently spread all over Asia and Oceania, given its average speed of propagation of 250 km/year in Europe [2] and even faster in Asia (550 km/year) [3]. To date, the disease has been considered the greatest threat for the pig industry worldwide [4]. Given the absence of a licensed vaccine against ASF, control efforts and surveillance implementation within country-specific eradication programs are of the utmost importance [5].

The disease’s etiological agent, ASF virus (ASFV), is a large, enveloped, double-stranded DNA virus, the only member of the *Asfarviridae* family [6]. The main target cells for ASFV replication are macrophages [7]. The virus genome size varies between ~170 and ~190 Kb in length, and contains 160–234 open reading frames (ORFs). Variabilities in the genome size are predominantly due to differences in the copy number of five different multigene families (MGF), which seem to play a central role in the control of host defenses [8]. ASFV was first identified in Kenya in 1921 [9] and, subsequently, several introductions from Africa to Europe have been described [10]. After the first case of virus incursion from Angola to Lisbon (1957), ASFV spread to other European, Caribbean and South American countries in 1960. Although the disease was successfully eradicated from all these territories, it has been circulating since 1978 in Sardinia, Italy [11]. After the first reported ASF case in Georgia in 2007, during the same year, the virus spread over the whole Caucasus region and the Russian Federation, and took 10 years to spread all over the European continent [12,13,14]. Since the first occurrence of ASF in mainland China in August 2018, the disease spread to Mongolia, Vietnam, Cambodia, Hong Kong, North Korea, Laos, Myanmar, Philippines, South Korea, Timor-Leste and Indonesia in less than one year. In 2020, new ASFV incursions were reported in Papua New Guinea and India [15,16].

In Sardinia, ASFV was first introduced in 1978, probably as a consequence of the import of contaminated food waste. The epidemiological dynamics of ASF in Sardinia involve three different *suidae* populations: domestic pigs, wild boar and illegal free-ranging pigs [17]. Several studies suggest a key role of free-range pigs in the persistence of ASFV in areas where they live in close contact with both domestic pigs and wild boar [11,18,19], thus acting as a virus reservoir [17,20]. The allegedly marginal role of wild boar in disease maintenance [19] has recently been demonstrated by Loi et al. [21]. In areas where the contact between suid populations is hindered by high biosecurity, the absence of illegal free-ranging pigs and well-planned surveillance, ASF has been reported to spontaneously fade out from wild boar [21,22]. Following the strict control measures against ASF put in place in Sardinia since 2015, a clear reduction in both virological and serological prevalence has been proven, and no outbreaks in domestic pigs or circulations in wild populations have been detected on the island since April 2019. Since then, 42 virus-negative but seropositive wild boars have been reported as outbreaks [23]. ASFV field strains are divided into 24 genotypes based on a fragment of the variable region of the B646L gene, which encodes for the major protein p72 [6,8,14]. All 24 of the genotypes have been detected in Africa, but only genotypes I and II have been found on other continents [24,25]. In particular, genotype II began to circulate in 2007 in Eastern Europe [26], reaching countries in Asia and Oceania only in the last two years [15]. Molecular studies have revealed that Sardinian ASFV belongs to *genotype I* (vp72) and p54 *genotype Ia* [27,28]. Differences were observed in the B602L gene, which is involved in viral morphogenesis, allowing differentiation between Sardinian isolates in two temporally related subgroups (X and III) [27,28]. The majority of the strains isolated from 1990 onwards (subgroup X) showed the deletion of 12–13 tetramers with respect to those isolated before 1990 (subgroup III). Sanna et al. [29] also reported an identical temporal subdivision of Sardinian ASF viruses into two subgroups differing via the deletion of a six-amino-acid repeat at the C-terminus of the CD2v protein encoded by the EP402R gene, which is characteristic of the strains isolated after 1990 [30]. Almost all of the Sardinian ASF viruses isolated after 1990 (modern strains) showed deletions in both the B602L and EP402R genes when compared to historical virus strains isolated before 1990 [29,30].

Global interest in ASF has drastically increased from 2014, along with an increasing number of ASFV sequences being published and used to identify genetic markers and trace routes of introduction through molecular epidemiology [31]. However, the publicly available ASFV WGSs are quantitatively and qualitatively insufficient, particularly considering the under-representation of the sylvatic cycle [31]. In total, 29 WGSs of *genotype II* (from ten affected countries) and 23 of *genotype I* (mostly from Sardinia) have been reported on in the last few years [20,31]. The first two WGSs of Sardinian isolates were obtained in 2016 [32,33]. Two more recent studies [20,34] analyzed a further 13 Sardinian ASFV full genomes, showing the remarkable genetic stability of these strains. The slow rate of ASFV molecular evolution, estimated from WGSs [34], represents a major limitation to the reconstruction of the evolutionary history of individual outbreaks spanning a limited period of time. It is unclear if this could be overcome by increasing the number of sequences sampled from a given outbreak, and how many sequences should be sampled to retrieve reliable evolutionary estimates. To answer these questions, we sequenced the complete genomes of 58 new ASFV isolates collected in Sardinia between 1978 and 2018; this large sequencing effort allowed us to depict accurately the phylogenetic structure of the ASF epidemic on the island. This study represents the first phylodynamic inference of ASFV based on the analysis of whole genomes. To the best of our knowledge, only Michaud et al. [35] and Alkhamis et al. [36] have performed phylodynamic investigations to obtain molecular dating and evolutionary estimates for African and Eurasian strains based on the sequencing of viral genes. Our study focuses for the first time on a closed epidemic system, applying phylodynamic methods to resolve the evolutionary history of ASFV on the Mediterranean island of Sardinia from 71 whole-genome sequences.

## 2. Materials and Methods

### 2.1. Sampling and Virus Isolation

**Figure 1:**
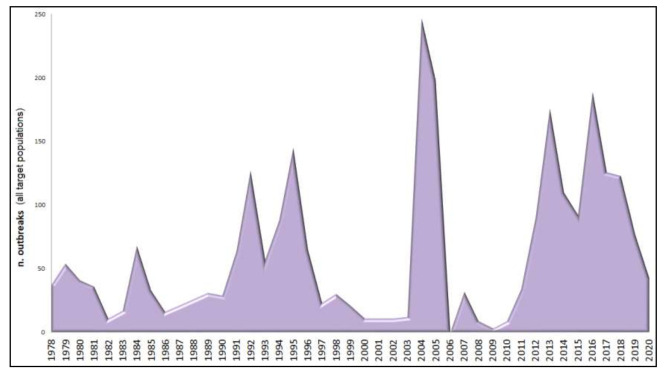
ASF outbreaks occurred in Sardinia in all the host populations of domestic pigs, wild boar, and free-ranging pigs between 1978 and 2020. Data are extracted from official reports recorded in the Italian National Information System for the Notification of Infectious Animal Disease (SIMAN) database, including both outbreaks confirmed for virus isolation and those for epidemiological correlation, according to the World Organization for Animal Health (OIE) Terrestrial Manual.

Between 1978 and 2018, a total of 2253 ASFV outbreaks were reported in Sardinia, both in domestic pig farms and wild boar [23]. The samples included in this analysis, all isolated from porcine monocytes/macrophages, have been selected from a virus archive consisting of about 400 strains stored in the laboratory of the Istituto Zooprofilattico Sperimentale della Sardegna (ASF Virus Archive, IZS of Sardinia, Sassari, Italy). We selected a total of 58 isolated (~15% of all samples available) according to the spatial and temporal distribution of the ASF outbreaks historically reported in Sardinia, placing emphasis on the four main epidemic peaks recorded during 1978–1984, 1990–1995, 2004–2005, and 2010–2018 (Figure 1), but also including at least 5% of the samples from each year of the epidemic.

**Figure 2:**
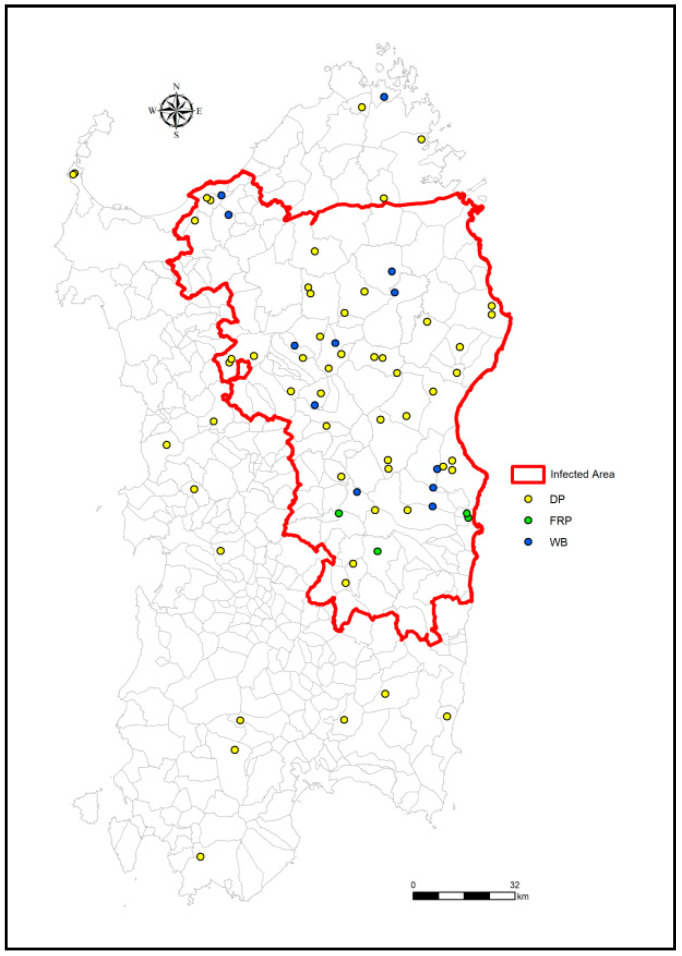
Spatial distribution of the 71 sampling sites in Sardinia. The yellow dots refer to domestic pig (DP) samples, the blue dots indicate wild boar (WB) samples, and green refer to illegal free-ranging pigs (FRP). The red line defines the limits of the historic wild boar infected area. The map reports both the 58 new and the 13 previously sequenced samples.

The resolution of the geographical location and collection date of the strains isolated from outbreaks between 1978 and 1995 are limited to province and year. To evaluate the spatial distribution of samples, these were collected based on the last cluster analysis performed by Rolesu et al. [23] to avoid a potential correlation between cases in wild boar and outbreaks notified in domestic pigs and to ensure homogeneous distribution within the historically infected area (Figure 2).

Sixteen samples were located outside the infected area. Therefore, to avoid possible bias, samples were drawn from the three host populations present in Sardinia (i.e., wild boar, domestic pigs and illegal free ranging pigs), as suggested by Forth et al. [31]. The presence of infectious ASFV was assessed using the Malmquist test (Haemoadsorption test) as described in the *OIE Terrestrial Manual* [37]. Tested samples were added to two-day-old porcine monocytes/macrophage monolayers, and cells were monitored daily for five days for haemoadsorption effects. In the presence of haemoadsorption, culture supernatant was collected and stored at −80 °C (ASF Virus Archive, IZS of Sardinia, Sassari, Italy). For negative samples, the Malmquist test was repeated by adding culture supernatants to fresh monocytes/macrophages; only after three negative results was the absence of live ASFV virus declared [37].

### 2.2. DNA Extraction, Quantification and Sequencing

Viral DNA was extracted from cell culture supernatant to perform the sequencing by Illumina platform using a QIAmp UltraSens Virus Kit (Qiagen, Hilden, Germany), following the manufacturer’s instructions. DNA quantification was performed using an Epoch microplate spectrophotometer (BioTek, Winooski, VT, USA) and a Qubit 2.0 Fluorometer (Thermo Fisher Scientific, Waltham, MA, USA), according to the manufacturer’s instructions. The whole sequence of 58 Sardinian viruses was obtained through Next Generation Sequencing (NGS). Viral DNA libraries were prepared using the Illumina Nextera XT DNA Sample preparation protocol (Illumina Inc., San Diego, CA, USA) and Nextera DNA Flex Library Prep kit (Illumina). Thirteen samples were sequenced with a HiSeq 2500 Instrument (Illumina) generating paired-end reads 2 × 150; the NGS of 44 samples was obtained using Novaseq 6000 (Illumina) generating paired-end reads 2 × 150 (at CRS4, Pula, Italy; and at AMES Group, Instrumental Polydiagnostic Center Srl, Naples, Italy, respectively). The sequences of the B602L (bases 96,322–97,938) and the EP402R genes (bases 68,928–70,112) were confirmed by Sanger sequencing using the primers and the methods described previously by Sanna et al. [29].

### 2.3. Bioinformatic Analysis

Genome data processing was performed using an in-house bioinformatic pipeline. The *bcl2fastq* program (https://support.illumina.com/sequencing/sequencing_software/bcl2fastq-conversion; last accessed date 30 June 2021) was used to convert BCL files generated by the sequencing systems to standard FASTQ file formats. *TrimGalore* (https://github.com/FelixKrueger/TrimGalore; last accessed date 30 June 2021) was used to quality trim the data and remove sequencing adaptors. The reads were aligned to the pig reference genome *Sus scrofa* version 10.2 [38], using the *bwa-mem* algorithm [39]. Only reads mapping uniquely to the ASFV genome were retained and re-aligned using *GEM* [40]. Aligned bam files were both sorted and indexed with *samtools* [41] and deduplicated with *Picard-tools* (https://broadinstitute.github.io/picard; last accessed date 30 June 2021). To obtain high-quality variants, *freebayes* [42] was used to call variants for each sample, using the KX354450 [33] sequence as the reference genome (parameters: “--ploidy 1 -X -u -m 20 -q 20 -F 0.2”). The assembly results were compared and analyzed using *MAFFT* 7.427 [43] and *Jalview* 2.10.3B.1 [44]; WGSs were aligned using *MAFFT* and polymorphism positions were visually checked using Jalview. Bam files of all virus samples were aligned against the KX354450 sequence and visually checked with *IGV* 2.4.14 [45]. The program *GC Content Calculator* (https://jamiemcgowan.ie/bioinf/gc.html; last accessed date 30 June 2021) was used to calculate the % G~C content. Genome annotation was performed using the *GATU software* [46], with KX354450 as the reference genome.

### 2.4. Genetic and Phylogenetic Diversity

As measures of diversity, we considered the average pairwise diversity among sequences (applied either to nucleotides only, or nucleotides and gaps, or whole insertions/deletions) and the number of variable sites (either considering only nucleotides, or nucleotides and gaps). The amount of phylogenetic information provided by additional sequences was defined via the classical Faith’s index of phylogenetic diversity [47], i.e., the overall tree length (sum of all branches up to the most recent common ancestor (MRCA) of the whole tree) computed on the neighbor-joining tree [48], estimated from the Hamming distance per base among sequences. The quantiles of the statistics for subsamples of a given size of the tree were computed from 100 random subsamples of the same size. All the statistics were implemented in *R* 4.1.0 (R Core Team, 2021) using the *ape* package [49].

### 2.5. Phylogeny, Molecular Dating and Evolutionary Rate

**Figure 3:**
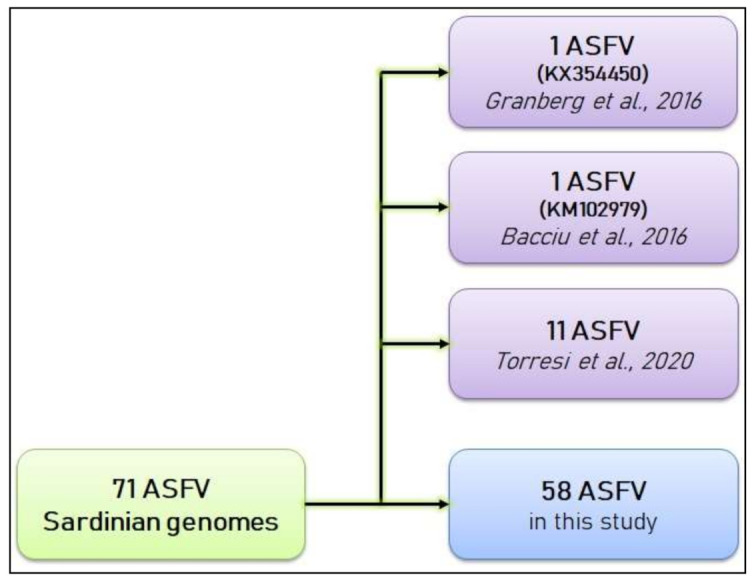
ASFV genome inclusion process flowchart. In total, 13 of the 71 genomes were derived from previous studies (Granberg et al., 2016; Bacciu et al., 2016; Torresi et al., 2020), and 58 are new genomes analyzed in this study.

As summarized in the ASFV flowchart (Figure 3), phylogenetic analyses were performed on a dataset composed of 71 WGSs of Sardinian ASFV, 13 of which were downloaded from *GenBank* and 58 were newly sequenced in the present study, along with three genomes (L60, E75 and Benin97) (Table 1, Table S1) belonging to genotype I and isolated outside the island, which showed the highest level of similarity with the previously described Sardinian genomes [20,32,33,34,50].

These latter three sequences represent strains from Africa and the Iberian Peninsula isolated between 1960 and 1997, included in the analysis as geographic outgroups. Genome alignments were carried out using the L-INS-I algorithm implemented in the software *MAFFT* 7.427 [43], and the obtained alignments were manually checked and edited using *Unipro UGENE* 35 [51]. To verify the reliability of the dataset obtained, the phylogenetic signal has been verified through the likelihood-mapping analysis of 10,000 random quartets by means of the software TreePuzzle [52]. This analysis allows to understand whether data are reliable for phylogenetic/phylodynamic inferences, or it suffers problems due to noisy data, alignment errors, homoplastic sites saturation and recombination [53].

The simplest evolutionary model that fits the sequence data best was detected using the software *JmodelTest* 2.1.7 [54]. In accordance with the best-fitting model, a Bayesian phylogenetic tree was obtained using the software *MrBayes* 3.2.7 [55] by setting the following model parameters: NST = 6, rates = invgamma, ngammacat = 4. Two independent runs, each consisting of four Metropolis-Coupled MCMC chains (one cold and three heated chains), were run simultaneously for 5 million generations, sampling trees every 1000 generations. The first 25% of the 5000 sampled trees were discarded as the burn-in. In order to verify the convergence of chains, we checked that the Average Standard Deviation of Split Frequencies (ASDSF) approached 0 [56], and the Potential Scale Reduction Factor (PSRF) was around 1 [54], following Scarpa et al. [57]. The phylogenetic tree was visualized and edited using *FigTree* 1.4.0 (available at http://tree.bio.ed.ac.uk/software/figtree; last accessed date 10 July 2021). 

The molecular dating of ASFV in Sardinia was performed using a Bayesian approach via the MCMC algorithm implemented in the software *BEAST* 1.8.2 [58], using the date of sample collection (year). In order to select the best model for dating inferences, both strict and uncorrelated log-normal relaxed clock models were tested under both parametric demographic (constant population size, exponential population growth, and expansion population growth) and piecewise-constant (Bayesian Skyline) models.

The demographic and clock models were selected by choosing the highest value of the natural logarithm of the Bayes Factor multiplied by two (2lnBF), using *Tracer* 1.7 [59] (as per Kass and Raftery [60]) with the modifications proposed by Suchard et al. [61]. 

After the selection of the best model, the phylogenetic time-scaled tree and the evolutionary rate were estimated running an MCMC of 100 million generations, sampling every 10,000 generations. The resulting log files were checked using the software *Tracer* 1.7 [59], ensuring that all parameters achieved an ESS (effective sample size) value of >200. A Maximum Clade Credibility (MCC) tree was reconstructed and visualized using the *TreeAnnotator* (Beast package) and *FigTree* software, respectively. 

The software *BEAST* 1.8.2 [58] was also used on different subsets of Sardinian ASFV genomes to estimate the evolutionary rate at different time points. These results were used to investigate temporal changes in the genetic diversity of ASFV in Sardinia using the software *Tracer* 1.7. The temporal dynamics of ASFV lineages were depicted by reconstructing the lineage-through-time plot. All phylogenetic/phylodinamic runs were carried out using the CIPRES Science Gateway [62] with a total effort of about 12,000 CPU hours of computation. Furthermore, in order to identify potential virus subgroups within the genetic clusters composed of isolates collected between 2002 and 2008 (*n* = 46), and to determine the dissimilarity represented by the genetic variability among genomes, a PCoA (principal coordinate analysis) was performed using GenAlEX 6.5 [63].

The possible occurrence of a statistically significant association between the genetic structuring by the phylogenetic analysis and the geographic origin of isolates was tested using the Pearson’s Chi-squared test by means of the stats package implemented in the R statistical environment (available at https://cran.r-project.org; last accessed date 18 September 2021). The same test has been also performed by computing a Monte Carlo simulation with 2000 replicates.

## 3. Results

### 3.1. Bioinformatic Analysis

Complete genome sequences were obtained for 40 ASFV isolates out of the 58 ASFV isolates analyzed in this study (Table 1, Table S1). The DNA sequences of these strains were 181,684–181,925 bp long, with GC contents ranging from 38.56 to 38.60% (Table S1). The inverted terminal repeats (ITRs), which included KP86R, KP96L, DP93R and DP86L genes, were missed at both ends, probably due to the difficulties encountered in assembling reads with low coverage. Annotation using the GATU software revealed a total of 231 ORFs (165 protein-coding genes and 66 uncharacterized reading frames or URFs). Of the 58 genomes, seven were annotated with GATU and deposited in GenBank, despite the presence of regions with low sequence quality. The presence of gaps in 11 sequences did not allow for annotation by GATU (identified via the SRR code in Table 1), and therefore these were deposited in the NCBI Short Read Archive (SRA). The median coverage obtained for all the sequences was between 250 and 6 (Table S2). The comparison of the Sardinian strains obtained in this study with all the ASFV genotype I complete genomes (Table 1) obtained from GenBank allowed for the detection of point mutations, including insertions or deletions (indels), as described in both Table S3 and Torresi et al.’s study [34].

### 3.2. Genomic Differences

The GenBank accession numbers, countries and years of isolation, virus genotypes, hosts and references for the strains analyzed in this work are reported in Table 1. We confirm the genome differences between Sardinian strains previously detected by Torresi in 2020 [34]; furthermore, we found other new point mutations. The genetic differences are summarized in Table S3. Ninety-five new point mutations were detected, located within several members of the MGF (MGF 360, MGF110 and MGF 505), and we also identified seven URFs (or undefined reading frames) and eleven intergenic regions (IGs). Some non-synonymous mutations were found in important regions coding for the following genes: A224L, a member of the inhibitors of apoptosis protein (IAP) family, able to inhibit caspase activity and cell death; A238L, an IkB-like protein gene with an important role in inhibiting the expression of transcription factors involved in host immune response [64,65]; NP868R, which encodes the capping enzyme [66]; D250R, an RNA polymerase that plays a role in the virulence of ASFV by altering or inhibiting the expressions of host proteins, through the degradation of mRNA by means of decapping the methylated cap attached to mRNA [67,68]; I329L, a structural protein involved in morphogenesis and the inhibition of IRF3 and NF-κB activation [65]; DP96R, involved in virus virulence and the inhibition of type I IFN expression and NF-κB [65]; EP402R (CD2 homolog), responsible for the adsorption of erythrocytes around infected cells (haemadsorption), which facilitates the spread of the virus in the host; B602L, a structural protein involved in morphogenesis (Table S3).

### 3.3. Impact of Improved Sampling on Genetic Diversity and Phylogenetic Resolution

**Figure 4:**
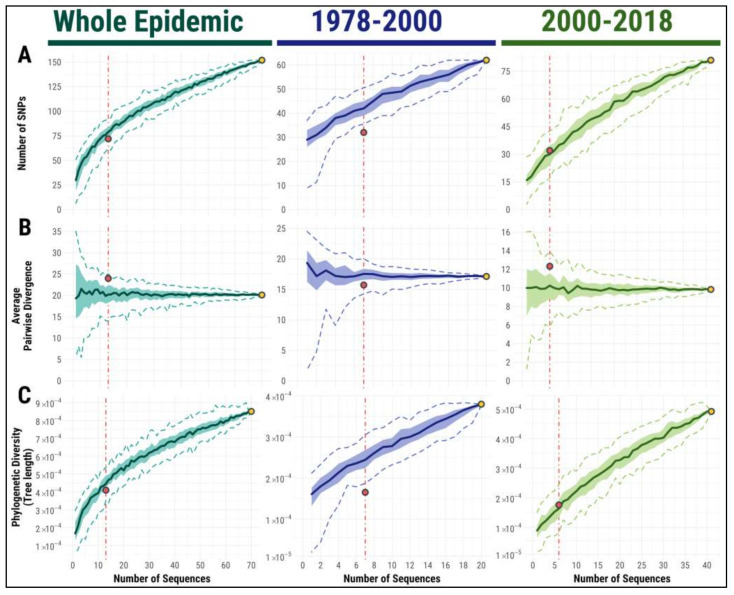
Dependence of genetic diversity and phylogenetic information on the number of ASFV sequences. Estimates of genetic diversity in terms of number of single nucleotide polymorphisms (SNPs) (**A**), average pairwise number of nucleotide differences among sequences (**B**), and phylogenetic diversity (**C**), as a function of the number of sequenced samples. The curves are computed for both the whole Sardinian epidemic, and for epoch 1 (1978–2000) and epoch 2 (2000–2018), separately. Dashed lines represent the 95% CI and ribbons represent the inter-quartile range for the estimates. The dots illustrate the values for all Sardinian sequences included in this work (in yellow) and for all previously published Sardinian sequences (in red).

The number of single nucleotide polymorphisms (SNPs) among the sequences estimates the genetic variability, and illustrates how much information on genomic diversity can be obtained from sampling a given number of sequences. The behavior of this measure with increasing numbers of sequences is illustrated in Figure 4A, and it is different between the first (pre-2000) and second halves (post-2000) of the epidemic.

In the first half of the epidemic, the number of variants detected from a few sequences was already quite high, and grew slowly but linearly with higher numbers of sequences. In the second half, the number of variants showed a rapid but sublinear increase with the number of sequences, as was similar for the whole epidemic. Sequencing between 10 and 20 sequences is sufficient to detect most common variants, but more and more variants are detected among additional sequences, albeit at a lower rate. Similar results are obtained when inserted/deleted bases are also considered (Figure S1).

Genetic diversity across the whole outbreak can be measured by the average divergence among pairs of sequences. This measure is expected to be stable with respect to sample size [69,70], and therefore offers a good way to estimate the local diversity, even from a few sequences. This is confirmed by our results (Figure 4B), suggesting that average pairwise divergence is the best measure for estimating and comparing the overall genetic diversity among ASFV outbreaks. However, it is worth noting that a small number of sequences can lead to both a high variance in the estimate and biased sampling, resulting in either an overestimate or underestimate of the actual diversity of the outbreak. For more than 10 sequences, the estimate becomes closer to its population value. 

Finally, we considered a measure that addresses phylogenetic resolution: the classical Field’s measure of phylogenetic diversity, estimated from the tree inferred from the sequences. Its behavior when sequences are added closely resembles the behavior of the number of variants, with a fast but slightly sublinear increase (Figure 4C). Over the 20–40 years of the epidemic, a decent phylogenetic reconstruction can be obtained with just 10–20 sequences, but even for an epidemic in a small geographical area, such as the Sardinian one, additional phylogenetic information remains in new sequences, even after sampling about two sequences per year. For the early phase of the epidemic, additional sequences provide less information, but there is no saturation effect, i.e., phylogenetic resolution continues increasing steadily with additional sequences.

### 3.4. Phylogenetic, Molecular Dating and Evolutionary Analyses

**Figure 5:**
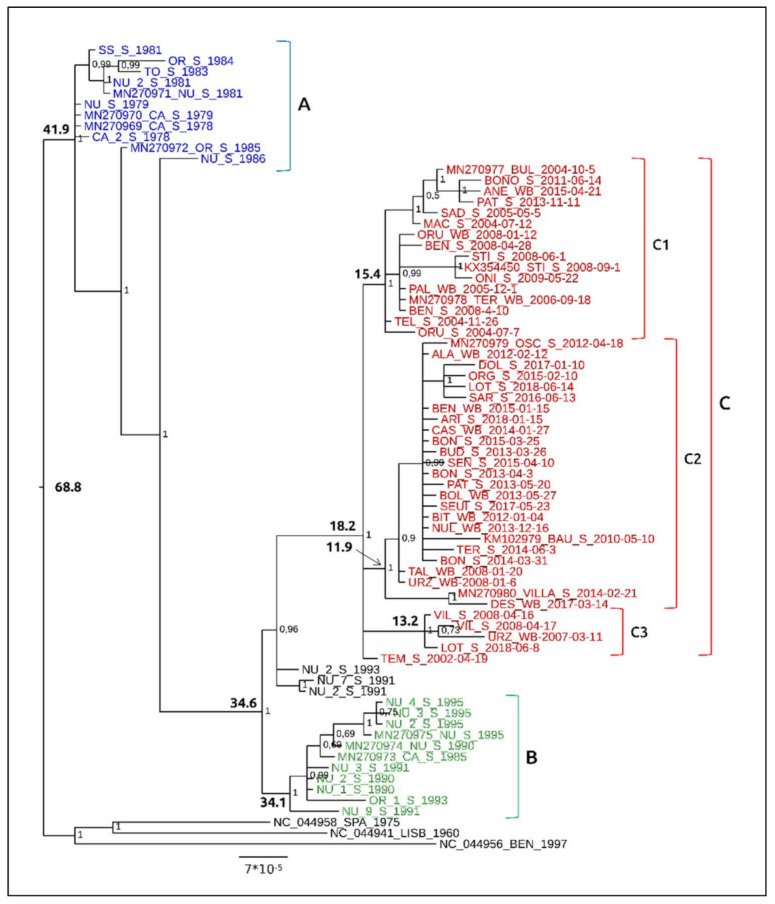
Bayesian phylogenetic tree based on the Sardinian ASFV genome dataset. Posterior probability values are reported for each node. The samples codes are as reported in Table 1.

The likelihood-mapping analysis indicates that the dataset obtained is reliable for phylogenetic/phylodynamic inferences, showing a strong phylogenetic signal. Indeed, the percentage of points in the network-like areas amounts to 11.6%, which is below the 20–30% threshold reported by Schmidt and von Haeseler (2012) [53] as indicative of datasets not reliable for phylogenetic analyses [52]. The phylogenetic information provided by the sequences in this study enabled us to reconstruct a detailed phylogenetic picture of the ASFV epidemic in Sardinia. The Bayesian phylogenetic tree (Figure 5) based on the whole dataset was mid-point rooted: nonetheless, the three viral strains from the Iberian Peninsula (Spain NC_044958, Portugal NC_044941) and Africa (Benin NC_044956), belonging to genotype I, were set as outgroups.

Indeed, these genomes grouped alone in a basal cluster. Within the ingroup of Sardinian genomes, viral sequences were spread across three groups (A, B and C), whose phylogenetic structuring is generally consistent with the date of collection of the viral isolates. No clear relationship between phylogenetic structuring and the geographic distribution of isolates was found. Indeed, the standard Pearson’s Chi-squared test and its Monte Carlo simulation, performed between all the clusters of the phylogenetic tree analysis, provided not significant values (with *p* = 0.60 and *p* = 0.59, respectively) indicating the lack of association between genetic variability and geographic origin of viral genomes. The Bayes Factor test revealed that the coalescent constant size, under the lognormal uncorrelated relaxed clock model, fitted data significantly better than other tested models (with 2lnBF = 24.856). Under this model, the phylogenetic time-scaled MCC tree showed that the MRCA to all viral genomes analyzed in the present study dated back to 68.8 years (95% HPD: 59.3–97.5) before 2018 (i.e., early 1949; 2018 corresponds to the collection year of the most recent isolated genome). The A group of sequences, derived from a common ancestor shared by all Sardinian genomes that dates back to about 41.9 years (95% HPD: 43.2–60.0) before 2018 (i.e., early 1976), was represented by a basal and heterogeneous group of viral genomes isolated between 1978 and 1986, likely representative of two large and divergent clades that occurred during that period. This puzzling evolutionary condition, if not directly attributable to a sampling bias, may be likely representative of landlocked lineages that went extinct without leaving descendants. A further highly inclusive group, whose origin dates back to 34.6 years (95% HPD: 33.937.2) before 2018 (i.e., early 1983), included the two main Sardinian clusters B and C, respectively. Cluster B, whose common ancestor dates back to 34.1 years (95% HPD: 33.3–35.8) before 2018 (i.e., late 1983), contained viral strains isolated between 1990 and 1995, along with one strain collected in 1985 in the south of the island (Cagliari). Cluster C, which originated 18.2 years (95% HPD: 17.6–24.3) before 2018 (i.e., late 1999), is included within the sister group of cluster B, and contains strains isolated from 2002 to 2018. Three viral strains, isolated in 1991 and 1993 from the middle of Sardinia (Nuoro), were set outside cluster C as possible landlocked lineages.

An internal well-supported sub-structuring was also found within the large cluster C, which contains three main sub-groups. Group C1, whose common ancestor dates back to 15.4 years (95% HPD: 15.4–20.1) before 2018 (i.e., middle 2002), and contains sequences isolated between 2004 and 2015, with 81% of strains isolated between 2004 and 2009; group C2, whose common ancestor dates back to 11.9 years (95% HPD: 10.3–16) before 2018 (i.e., early 2006), includes sequences isolated between 2008 and 2018, with 88% of strains collected between 2012 and 2018; group C3, whose common ancestor dates back to 13.2 years (95% HPD: 11.6–16.3) before 2018 (i.e., late 2004), includes four sequences isolated in 2007, 2008 and 2018. A further single genome, isolated from the north of the island (Tempio) in 2002, was also included in cluster C outside the three internal sub-groups. 

The sub-structuring within cluster C was confirmed by PCoA (Figure S2), the results of which exactly matched the topology of the phylogenetic tree.

Further evolutionary inferences on the spread of ASFV in Sardinia were also made based on the analysis of a dataset grouping only genomes from the island.

**Figure 6:**
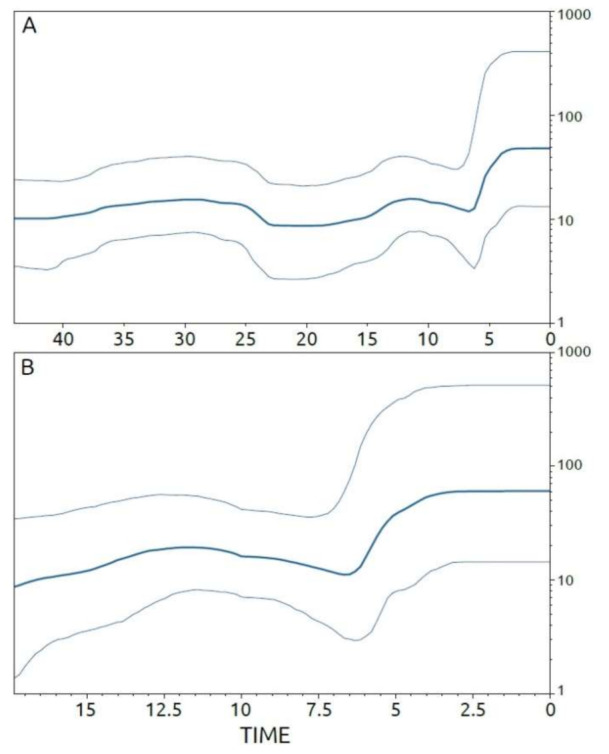
Bayesian skyline plots depicting the evolutionary history of ASFV on the Mediterranean island of Sardinia: whole Sardinia dataset and cluster C. The viral effective population size (*y*-axis) is shown as a function of time (*x*-axis), with the units expressed in years. (**A**) ASFV whole Sardinia dataset, (**B**) dataset encompassing only Sardinian sequences included in cluster C of the phylogenetic tree. The bold lines denote the median population estimates and the grey lines denote the 95% high posterior density (HPD) region.

Under the same model (lognormal uncorrelated relaxed clock model), the viral evolutionary clock of the ASFV strains evolving in Sardinia between 1978 and 2018 was estimated to be 3.49 × 10^−6^ substitutions/site/year (95% HPD 2.53 × 10^−6^–4.58 × 10^−6^). Temporal patterns of viral spreading, inferred from the Bayesian skyline plot (BSP), were calculated not only for the whole Sardinian dataset, but also for the viral strains included in cluster C of the phylogenetic tree, and for the largest of its internal sub-groups (C1 and C2) (Figure 5 and Figure 6). 

**Figure 7:**
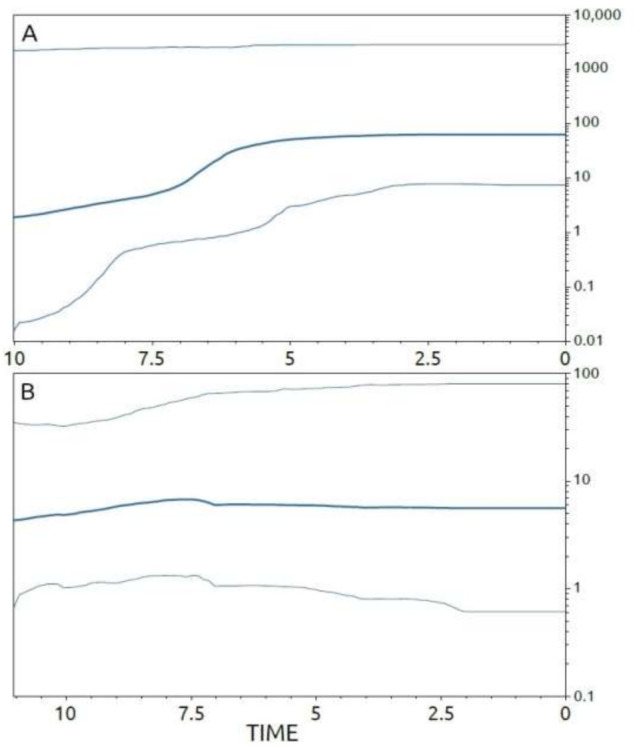
Bayesian skyline plots depicting the evolutionary history of ASFV on the Mediterranean island of Sardinia: sub-groupss C2 and C1. The viral effective population size (*y*-axis) is shown as a function of time (*x*-axis), with the units expressed in years. (**A**) Dataset encompassing only Sardinian sequences included in sub-group C2 of the phylogenetic tree; (**B**) dataset encompassing only Sardinian sequences included in sub-group C1 of the phylogenetic tree. The bold lines denote the median population estimates and the grey lines denote the 95% high posterior density (HPD) region.

The patterns in the virus’ demographic history, reconstructed by the BSP (Figure 6A) using all Sardinian isolates, show a general consistency in the expansion size of ASFV populations, starting from about 42 years before 2018 and reaching about six years before 2018 (i.e., from 1976 to 2012), after which a short-term increase in viral diversity was seen. This increase lasted about three years (i.e., from 2013 to 2016) and was followed by a new phase of consistency in the expansion of the viral population’s size. The BSP calculated for cluster C shows a similar pattern that described using the whole Sardinian dataset (Figure 6B). This similarity is also confirmed by the evolutionary rate of cluster C, which matches the value calculated for the whole Sardinian sample, estimated in the order of 3.20 × 10^−6^ substitution/site/year (95% HPD of 2.04 × 10^−6^–4.30 × 10^−6^). A similar pattern was also found for sub-group C2 (Figure 7A) (molecular clock of 3.19 × 10^−6^ substitution/site/year; 95% HPD 6.09 × 10^−7^–5.87 × 10^−6^), which showed a comparably consistent initial expansion in the size of the viral population, with a weak increase occurring six years before 2018 (i.e., 2012) that was immediately followed by a renewed consistency in the size expansion. On the contrary, the patterns of viral spread analyzed for sub-group C1 (Figure 7B) (molecular clock of 3.38 × 10^−6^ substitution/site/year; 95% HPD 1.03 × 10^−6^–5.92 × 10^−6^) suggest consistency in the size expansion of ASFV populations, which continued uninterrupted up to 2018.

**Figure 8:**
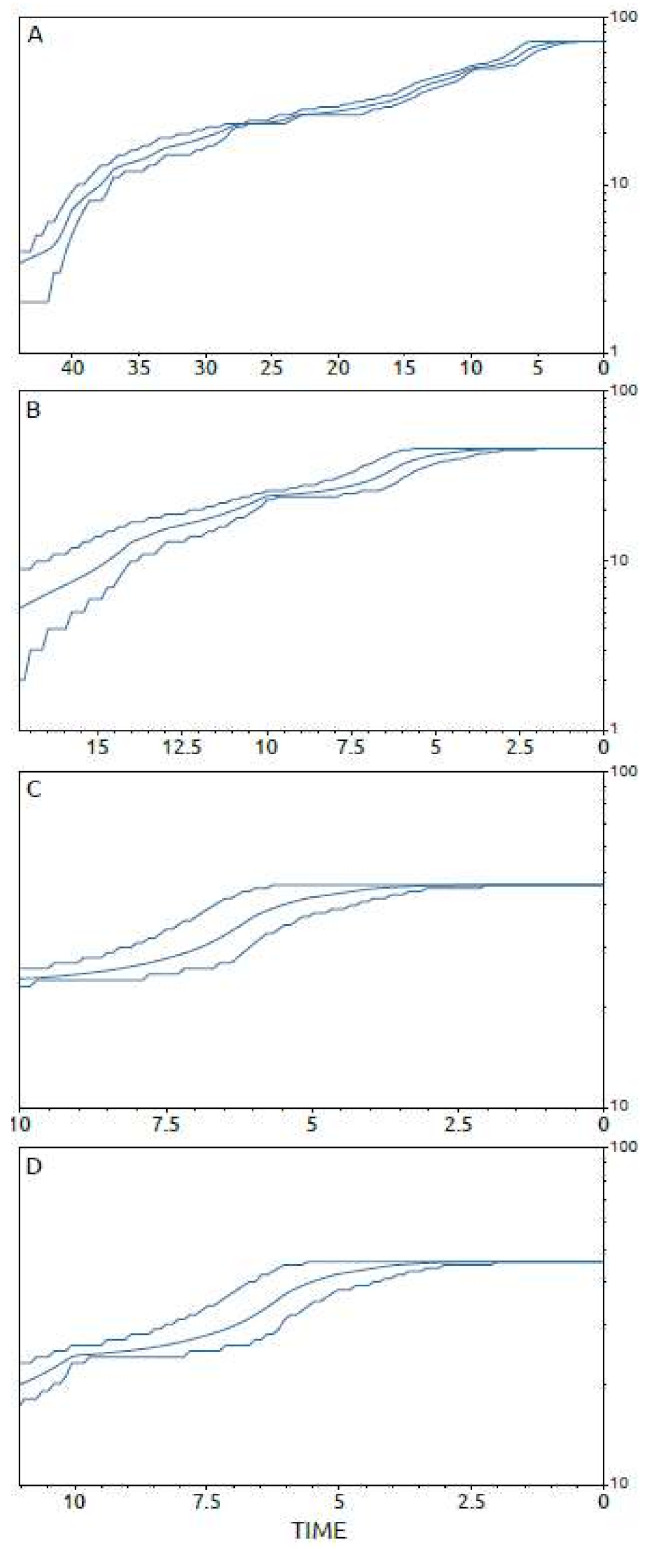
Number of ASFV lineages through time. Logarithmic expansion in the amount of viral lineages (*y*-axis) is shown as a function of time from the present (*x*-axis), with the units expressed in years. (**A**) ASFV whole Sardinia dataset; (**B**) dataset encompassing only Sardinian sequences included in cluster C of the phylogenetic tree; (**C**) dataset encompassing only Sardinian sequences included in sub-group C2 of the phylogenetic tree, and (**D**) dataset encompassing only Sardinian sequences included in sub-group C1 of the phylogenetic tree. The bold lines denote the median estimates and the grey lines denote the 95% high posterior density (HPD) region.

The amount of viral lineages in Sardinia (Figure 8A) increased strongly and consistently from the origin of the whole Sardinian ASFV clade up to five years before 2018 (i.e., 2013), when it stopped. Consistent results showing almost identical trends in the increase in lineages were also obtained for cluster C and its sub-groups C2 and C1 (Figure 8B–D).

## 4. Discussion

In this study, we analyzed 71 whole-genome sequences from ASFV isolates collected during a 40-year period of ASF endemicity in Sardinia. The geographic distribution of such samples can be considered as representative of the historically endemic ASF areas [21,71,72,73]. This Mediterranean island is the oldest endemic area of Europe, and is characterized by a unique epidemiological context: ASFV circulates and replicates between and within populations of domestic pigs, free-ranging pigs and wild boar, without the presence of *Ornithodoros* ticks [11,17,18,19,20,21,71,72,74]. Both the 40 years of isolation and the absence of other genotypes make this context ideal for investigating patterns of ASFV evolutionary transmission in a closed epidemiological system, with (presumably) no recent introductions from outside. The Sardinian samples selected for the analyses in this study perfectly meet the requirements for a complete and unbiased whole-genome sequencing analysis, including samples from all the involved porcine populations [31,36] and representing, to the best of our knowledge, the largest ASFV sample size ever studied at the whole-genome level. The large number of newly sequenced samples generated by the Sardinian epidemic provides a unique testing ground to assess the impact of sampling density and the number of sequences. In theory, increasing the number of sequences of a slowly evolving virus could have an intrinsically limited impact on phylogenetic resolution because the new sequences would be too similar to existing ones. Instead, this study provides evidence of how, in ASFV outbreaks, sequencing more samples leads to an improved resolution of genetic and phylogenetic diversity [31]. To understand the quantity of information provided by each additional sampled sequence, we computed some measures of genetic variability for random subsets of sequences of different sizes, and explored how they vary with the addition of increasing numbers of sequences derived from the epidemic. Considering that the evolution of ASFV is quite slow, even for an epidemic that lasts decades, the sequencing of an increasing number of ASFV samples provides more detailed genomic and phylogenetic information, and reveals finer details of the phylogenetic structure, in turn enhancing the phylodynamic tools used to study the evolution and epidemiology of the virus [73,75]. These results are promising for the future of ASFV phylodynamic studies. In the immediate future, our results will support the push for concerted sequencing efforts focused on outbreaks of ASFV genotype II in other Eurasian regions; sequences from these outbreaks are expected to harbor a wealth of phylodynamic information, especially considering their wide geographical spread.

The availability of WGSs revealed potentially relevant genetic variations that could have caused differences in virus fitness. Compared with the ASFV genomes analyzed by Torresi et al. [34], we found new point mutations, some of which are exclusive to single strains. Differences were observed in genes encoding for RNA, DNA polymerases, transcription factors, and the proteins involved in host immune response, morphogenesis and virus virulence. Other point mutations occurred in genes coding for proteins with unknown functions and undefined reading frames (or URFs). New and already known indels affecting several genes created frameshifts and consequent changes in protein length.

However, the effects of these changes on protein function are unknown. New mutations arose in a total of 25 isolates (35.2%) sampled from 1983 to 2018, and these were unique to and typical of landlocked strains. This could be due to the control measures adopted following the eradication plans enforced since 1982.

The evolutionary rate of ASFV as estimated in the present work, based on the analysis of 71 complete genomes isolated in Sardinia, was consistent with the value found by Torresi et al. [34] using a smaller number of Sardinian samples (*n* = 14), thus suggesting that the estimates so far obtained are likely representative of the real mutational rate of this virus in the island. The substitution rate found for ASFV in Sardinia is two orders of magnitude lower than the value calculated by Alkhamis et al. [36] for outbreaks in Eurasia and Africa between 1960 and 2015. However, such a finding should be explained considering that Alkhamis et al. [36] analyzed a dataset composed of 96 vp72-CVR sequences belonging to different genotypes from different countries, removing clones and sequences with no phylogenetic structure from the 665 strains available in GenBank. The use of whole-genome sequencing technology on such a large number of Sardinian ASFV isolates in this study does not allow a direct comparison between these two evolutionary rates. Furthermore, the predominant type of smallholding farms, free-ranging breeding, the general rural context, the insularity of Sardinia, the limitations on pig trading imposed under the measures against ASF and the high selective pressure caused by the culling of pigs following each detected ASF outbreak could have placed constraints on both the expansion and the evolution of the virus, thus affecting its evolutionary pattern in this small Mediterranean area. 

The common ancestor of all the Sardinian viral genomes analyzed in the present study dates back to 1976, thus post-dating the previous estimate provided by Torresi et al. (1954) by about 22 years [34]. The discrepancy between the two values may be due to the different clock and demographic models used to perform the analyses, and to the different numbers of Sardinian genomes analyzed in the two studies. Here, 58 additional new ASFV genomes were included in the analysis, thus increasing the resolution by adding either 80% more Sardinian sequences or 52.2% more Eurasian ASFV genomes. We also tried to reduce the possibility of bias in sample coverage over the same period analyzed by Torresi et al. [34] (1978–2014) by selecting genomes according to the spatial and temporal distribution of the ASF outbreaks. In addition, the larger number of isolated genomes that we used were distributed over a period that was four years longer, which might have permitted finer molecular dating. 

In this context, the different molecular dates of Sardinian strains found in the present study (i.e., 1976 as the MRCA) align with the third historical African wave reported in 1964 [76], and the emergence of the disease in the Iberian Peninsula between 1960 and 1975 [77,78], which led to the incursion of ASFV into both Malta and Sardinia in 1978. These dates are further consistent with the findings of Alkhamis et al. [36], who confirmed that common ancestors of ASFV isolated from Eurasia and West Africa (the ancestors of viruses circulating in the Iberian Peninsula, and consequently of Sardinian ASFVs) were about 87 and 68 years more recent, respectively, than those isolated in East Africa.

The genetic diversity and the diversification rate of ASFV in Sardinia steadily increased between its first report in 1978 and the first decade of the 2000s; accordingly, we identified a constant trend in the growth of viral lineages, which only contracted after 2013. Such a result likely reflects the trend of diversification following the establishment of a circulating viral population in a small number of genetically similar founders during their adaptation to new habitats [79], suggesting that the Sardinian ASFV population may have reached an equilibrium in 2013. This hypothesis is in line with a recent network analysis that aimed to estimate the number of secondary cases in Sardinia, which identified the maximum reproduction number (R_0_) in June 2013 (8.7, 95% CI = 2.6–9.1) and a gradual decrease until 2017, when the R_0_ ≤ 1 (indicating that ASFV circulation faded out) [23]. This could explain the increase in the genetic variability of the virus population. Accordingly, during this 35-years process of adaptation, the spreading of the viral population among hosts followed a trend of constant size expansion up to 2012, at which point a peculiar trend emerged; between 2012 and 2013, the diffusion of ASFV among hosts quickly increased, before returning to consistency.

Moreover, the stronger disease control measures applied in 2014 cannot be ignored as potential factors in the process of viral spreading. The central role of free-ranging pigs in ASFV transmission has previously been described [11,20,80]. The fight against these illegally bred animals started in 2014, and culling (officially started in 2016) drastically reduced the susceptible population. This effect was even more evident in the main clusters detected by Rolesu et al. [23] in inner Sardinian areas, where free-ranging pigs and wild boar shared a habitat that was closer to domestic pig farms.

The genetic variability highlighted via the analysis of the ASFV genomes isolated in Sardinia over the 40-year period of endemicity was consistent with the results obtained by Torresi et al. [34]. Furthermore, three main genetic groups were identified, differentiated on a temporal basis, which clearly reflect the ASF epidemic’s trend previously reported in susceptible populations [23]. 

The oldest Sardinian genetic group found in the present study includes viral strains that were likely differentiated outside Sardinia in 1976. The viral strains within this group are representative of the strains that generated the first wave of the ASF epidemic in Sardinia between 1978 and 1986. Even though all the available viral isolates associated with the ASFV waves during the 1980s were analyzed, the low number of available sequences precluded a full understanding of the evolutionary dynamics of the virus during the first few years of its spread in the island. However, a divergence between the strain that reached Sardinia in 1978 and those characterizing ASFV infections during the mid-1980s may be hypothesized, in accordance with the results here obtained.

The genetic group including viral strains isolated during the first half of the 1990s in the endemic area in the middle of the island is likely representative of the second wave of the ASF epidemic in Sardinia (Figure 1).

The high number of genomes available for the most recent genetic group (cluster C of the phylogenetic tree analysis), which includes 57% of the Sardinian sequences analyzed, along with the detailed information regarding the collection dates of samples, permit a detailed overview of the last epidemic in the susceptible Sardinian population during the 2000s.

Although the high number of ASFV genomes in this group would allow for proving the presence of geographic structuring among viral strains if any, no relevant differentiation among Sardinian areas was found, possibly because of the constant viral gene flow throughout the sampling sites. This is also supported by the long-transmission patterns identified via a network analysis of the same area [23].

The lack of association between genetic variability and geographic origin is also supported by the long-transmission patterns identified via a network analysis performed for the same area [23]. In addition, it should be also taken into account that the ASFV genotypes I circulating in Sardinia were supposed to evolve from the same ancestors and no other viruses were introduced in the island [17].

It is important to note that the association between genetic structuring and the diffusion of ASFV among domestic and free-ranging pigs and wild boar populations was not tested in the present study as the biases described in Loi et al. [17] would have strongly affected results. Indeed, domestic and free-ranging pigs actually represent the same populations differently bred, which are genetically distinguishable by wild boar. ASFV isolates started to be defined as “from domestic or free-ranging pigs” by Veterinary Services declaration during the stamping out in 2019 [80], and in this context, since culling action on free-ranging pigs in Sardinia started in 2016, the distinction between domestic and free-ranging pigs is not applicable for the isolates included in our dataset that were collected in the previous years. Furthermore, recent studies provided many scenarios of ASF transmission dynamics, describing the central role of free-ranging pigs as the ASFV reservoir and the secondary role played by wild boar [11,20,21].

In addition, in the present study, the analysis to assess the level of association between genetic structuring and the diffusion of ASFV among hosts might have been further affected by the discrepancy between the number of wild boar and pigs which were sampled to isolate viral genomes. Indeed, the sampling plan reflects the numbers of outbreaks which occurred in the island where virus isolation was feasible.

The evolutionary trend of ASFV in Sardinia is generally constant and relatively slow, and the genetic variability found in the present study is likely shaped by specific human-mediated activities (i.e., animal movements, hunting management, outdoor animal breeding) [23]. Our findings corroborate the assumption that Sardinia is a closed and isolated system of ASFV dispersal, without any recent external contributions from the continent. Consistently, in Africa, animal trade peaks are considered to be directly associated with the development of evolutionary patterns of this virus [36].

However, when interpreting phylogenetic results for ASFV genomes, it should be taken into consideration the possible occurrence of homoplastic features within the analyzed dataset, which may introduce biases due to homologous recombination and positive selection [81,82,83]. Indeed, the variability arisen from homoplasious variation and evolutionary selection, which is not consistent with vertical inheritance, may affect results (Farlow et al., 2018). Anyhow, to verify the reliability of the dataset used for phylogenetic inferences in the present study, the phylogenetic signal has been tested using the likelihood-mapping analysis. Results pointed out a strong phylogenetic signal, which is indicative of a reliable dataset suitable for phylogenetic/phylodynamic inferences [53]. In conclusion, this study corroborates the importance of using a whole-genome phylodynamic-based approach to infer the demographic patterns of ASFV. In the future, this methodology should be applied to disentangle the evolutionary histories of viruses when trying to understand how human actions (i.e., implementation of infected areas, stamping out, and movement restriction) influence their potential for dispersal and adaptation to new hosts.

## Figures and Tables

**Table 1:** Metadata associated with the ASFV whole genomes analyzed in this study. GenBank accession number and tip labels used in the phylogenetic trees are also reported. DP: domestic pig; WB: wild board; FRP: free-ranging pig.

Sample ID	Accession Number	Country of Origin	Collection Date	Host	P72 Genotype	Tree Label	Methods	Reference
L60	NC044941	PORTUGAL	1960	DP	I	NC_044941_LISB_1960	Sanger	De Villiers et al., 2016
E75	NC044958	SPAIN	1975	DP	I	NC_044958_SPA_1975	Sanger	Portugal et al., 2015
56/Ca/1978	MN270969	CAGLIARI	1978	DP	I	MN270969_CA_S_1978	Illumina_Sanger	Torresi et al., 2020
CA1978_2	MW723480	CAGLIARI	1978	DP	I	CA_2_S_1978	Illumina_Sanger	This study
57/Ca/1979	MN270970	CAGLIARI	1979	DP	I	MN270970_CA_S_1979	Illumina_Sanger	Torresi et al., 2020
NU1979	MW723481	NUORO	1979	DP	I	NU_S_1979	Illumina_Sanger	This study
139/Nu/1981	MN270971	NUORO	1981	DP	I	MN270971_NU_S_1981	Illumina_Sanger	Torresi et al., 2020
NU1981_2	SRR13976567	NUORO	1981	DP	I	NU_2_S_1981	Illumina_Sanger	This study
SS1981	MW788409	SASSARI	1981	DP	I	SS_S_1981	Illumina_Sanger	This study
ITALY1983	SRR13976568	TORINO	1983	DP	I	TO_S_1983	Illumina_Sanger	This study
OR1984	MW800838	ORISTANO	1984	DP	I	OR_S_1984	Illumina_Sanger	This study
140/Or/1985	MN270972	ORISTANO	1985	DP	I	MN270972_OR_S_1985	Illumina_Sanger	Torresi et al., 2020
85/Ca/1985	MN270973	CAGLIARI	1985	DP	I	MN270973_CA_S_1985	Illumina_Sanger	Torresi et al., 2020
NU1986	MW723482	NUORO	1986	DP	I	NU_S_1986	Illumina_Sanger	This study
141/Nu/1990	MN270974	NUORO	1990	DP	I	MN270974_NU_S_1990	Illumina_Sanger	Torresi et al., 2020
NU1990_1	MW723482	NUORO	1990	DP	I	NU_1_S_1990	Illumina_Sanger	This study
NU1990_2	SRR13976561	NUORO	1990	DP	I	NU_2_S_1990	Illumina_Sanger	This study
NU1991_2	MW723484	NUORO	1991	DP	I	NU_2_S_1991	Illumina_Sanger	This study
NU1991_3	MW723485	NUORO	1991	DP	I	NU_3_S_1991	Illumina_Sanger	This study
NU1991_7	MW723486	NUORO	1991	DP	I	NU_7_S_1991	Illumina_Sanger	This study
NU1991_9	SRR13976566	NUORO	1991	DP	I	NU_9_S_1991	Illumina_Sanger	This study
OR1993_1	MW723487	ORISTANO	1993	DP	I	OR_1_S_1993	Illumina_Sanger	This study
NU1993_2	MW723488	NUORO	1993	DP	I	NU_2_S_1993	Illumina_Sanger	This study
142/Nu/1995	MN270975	NUORO	1995	DP	I	MN270975_NU_S_1995	Illumina_Sanger	Torresi et al., 2020
NU1995_2	MW723489	NUORO	1995	DP	I	NU_2_S_1995	Illumina_Sanger	This study
NU1995_3	MW723490	NUORO	1995	DP	I	NU_3_S_1995	Illumina_Sanger	This study
NU1995_4	MW723491	NUORO	1995	DP	I	NU_4_S_1995	Illumina_Sanger	This study
BENIN_1997	NC044956	BENIN/AFRICA	1997	DP	I	NC_044956_BEN_1997	/	Chapman et al., 2008
24225_2002	MW788411	TEMPIO	2002	DP	I	TEM_S_2002-04-19	Illumina_Sanger	This study
26/Ss/2004	MN270977	BULTEI	2004	DP	I	MN270977.1_BUL_2004-10-5	Illumina_Sanger	Torresi et al., 2020
44076_2004	MW723500	ORUNE	2004	DP	I	ORU_S_2004-07-7	Illumina_Sanger	This study
45539_2004	SRR13976563	MACOMER	2004	DP	I	MAC_S_2004-07-12	Illumina_Sanger	This study
74377_2004	MW723496	TELTI	2004	DP	I	TEL_S_2004-11-26	Illumina_Sanger	This study
72407_2005	MN270978	TERGU	2005	DP	I	MN270978_TER_WB_2006-09-18	Illumina_Sanger	Torresi et al., 2020
22649_2005	MW723497	SADALI	2005	DP	I	SAD_S_2005-05-5	Illumina_Sanger	This study
72398WB_2005	MW723495	PALAU	2005	WB	I	PAL_WB_2005-12-1	Illumina_Sanger	This study
72912WB_2007	MW723498	URZULEI	2007	WB	I	URZ_WB-2007-03-11	Illumina_Sanger	This study
47/Ss/2008	KX354450	STINTINO	2008	DP	I	KX354450_STI_S_2008-09-1	Pacbio	Granberg et al., 2016
1537WB_2008	MW788405	URZULEI	2008	WB	I	URZ_WB-2008-01-6	Illumina_Sanger	This study
4996WB_2008	MW723492	TALANA	2008	WB	I	TAL_WB_2008-01-20	Illumina_Sanger	This study
23221_2008	MW723494	VILLASOR	2008	DP	I	VIL_S_2008-04-17	Illumina_Sanger	This study
25185_2008	MW788410	BENETUTTI	2008	DP	I	BEN_S_2008-04-28	Illumina_Sanger	This study
22943_2008	MW788406	VILLASOR	2008	DP	I	VIL_S_2008-04-16	Illumina_Sanger	This study
22137_2008	MW723499	BENETUTTI	2008	DP	I	BEN_S_2008-4-10	Illumina_Sanger	This study
46830_2008	MW723493	STINTINO	2008	DP	I	STI_S_2008-06-1	Illumina_Sanger	This study
28170_2009	SRR13976565	ONIFERI	2009	DP	I	ONI_S_2009-05-22	Illumina_Sanger	This study
1628_2009	SRR14601691	ORUNE	2009	DP	I	ORU_S_2008-12-01	Illumina_Sanger	This study
26544/OG10	KM102979	BAUNEI	2010	DP	I	KM102979_BAU_S_2010-05-10	Illumina_Sanger	Bacciu et al., 2016
31208_2011	MW736612	BONO	2011	DP	I	BONO_S_2011-06-14	Illumina_Sanger	This study
63525WB_2012	MW736603	BITTI	2012	WB	I	BIT_WB_2012-01-04	Illumina_Sanger	This study
97/Ot/2012	MN270979	OSCHIRI	2012	DP	I	MN270979_OSC_S_2012-04-18	Illumina_Sanger	Torresi et al., 2020
2019WB_2012	MW736598	ALA’ DEI SARDI	2012	WB	I	ALA_WB_2012-02-12	Illumina_Sanger	This study
30322_2013	MW736600	BUDDUSO’	2013	DP	I	BUD_S_2013-03-26	Illumina_Sanger	This study
32516_2013	MW736607	BONORVA	2013	DP	I	BON_S_2013-04-3	Illumina_Sanger	This study
47039_2013	MW736597	PATTADA	2013	DP	I	PAT_S_2013-05-20	Illumina_Sanger	This study
49179WB_2013	MW736601	BOLOTANA	2013	WB	I	BOL_WB_2013-05-27	Illumina_Sanger	This study
98039_2013	MW736599	PATTADA	2013	DP	I	PAT_S_2013-11-11	Illumina_Sanger	This study
113049WB_2013	MW736608	NULVI	2013	WB	I	NUL_WB_2013-12-16	Illumina_Sanger	This study
22653/Ca/2014	MN270980	VILLANOVATULO	2014	DP	I	MN270980_VILLA_S_2014-02-21	Illumina_Sanger	Torresi et al., 2020
11484WB_2014	SRR13975654	CASTELSARDO	2014	WB	I	CAS_WB_2014-01-27	Illumina_Sanger	This study
35479_2014	MW788408	BONORVA	2014	DP	I	BON_S_2014-03-31	Illumina_Sanger	This study
51268_2014	MW736605	TERGU	2014	DP	I	TER_S_2014-06-3	Illumina_Sanger	This study
6396WB_2015	MW736609	BENETUTTI	2015	WB	I	BEN_WB_2015-01-15	Illumina_Sanger	This study
15998_2015	MW736604	ORGOSOLO	2015	DP	I	ORG_S_2015-02-10	Illumina_Sanger	This study
28928_2015	MW736610	BONORVA	2015	DP	I	BON_S_2015-03-25	Illumina_Sanger	This study
31479_2015	MW788407	SENNORI	2015	DP	I	SEN_S_2015-04-10	Illumina_Sanger	This study
33747WB_2015	MW736613	ANELA	2015	WB	I	ANE_WB_2015-04-21	Illumina_Sanger	This study
53706_2016	MW736602	SARULE	2016	DP	I	SAR_S_2016-06-13	Illumina_Sanger	This study
3312_2017	SRR13976564	DOLIANOVA	2017	DP	I	DOL_S_2017-01-10	Illumina_Sanger	This study
34403WB_2017	MW736606	DESULO	2017	WB	I	DES_WB_2017-03-14	Illumina_Sanger	This study
52060_2018	SRR13976569	SEUI	2017	FRP	I	SEUI_S_2017-05-23	Illumina_Sanger	This study
8343_2018	SRR13976571	ARITZO	2018	FRP	I	ARI_S_2018-01-15	Illumina_Sanger	This study
54684_2018	MW647171	LOTZORAI	2018	FRP	I	LOT_S_2018-06-8	Illumina_Sanger	This study
56140_2018	MW736611	LOTZORAI	2018	FRP	I	LOT_S_2018-06-14	Illumina_Sanger	This study

## Data Availability

All data are reported in the main text and Supplementary Materials.

## Supplementary Materials

The following are available online at https://www.mdpi.com/article/10.3390/v13101994/s1, Figure S1: Dependence of genetic diversity with indels on the number of ASFV sequences. Estimates of genetic diversity in terms of number of polymorphic sites, counting both nucleotides and gaps due to indels (A), average pairwise number of nucleotide differences among sequences counting both nucleotides and gaps (B), and average pairwise number of indels differing among sequences, as a function of the number of sequenced samples (C). The curves are computed both for the whole Sardinian epidemic and for epoch 1 (1978–2000) and epoch 2 (2000–2018) separately. Dashed lines represent the 95% CI and ribbons represent the inter-quartile range of the estimates. The dots illustrate the values for all Sardinian sequences included in this work (in yellow) and for all previously published Sardinian sequences (in red). Figure S2: PCoA for a sub-group of Sardinian ASFV genomes. Analysis of principal coordinates of ASFV genomes included in cluster C of the phylogenetic tree in Figure 3. The sample’s codes are reported in Table 1. In the plot, the spots representing some of the sequences included in the analyzed dataset are graphically overlapped. Table S1. Accession number, total length and GC% content of 40 African swine fever virus strains sequenced in this study. Table S2. Samples coverage and diagnostic centre of the strains sequenced in this study. Table S3. Genetic mutations identified in Sardinian viruses isolated from outbreaks reported between 1978 and 2018. SNPs and indels positions were obtained from the aligning of the Sardinian sequences to the KX354450 ASFV strain. Mutations were designated from M1 to M95.
