# Supplementary material for: A Deeper Insight into Evolutionary Patterns and Phylogenetic History of ASFV Epidemics in Sardinia (Italy) through Extensive Genomic Sequencing
Source: Viruses. 2021 Oct 4;13(10):1994. doi: 10.3390/v13101994 (PMC8539718; doi:10.3390/v13101994)
Supplement: Supplementary file 1 [file viruses-13-01994-s001.zip › S1 Table.pdf]

S1 Table. Accession number, total length and GC% content of 40 African swine fever virus strains sequenced in this study

Sample ID	Accession Number	Total Lenght	GC%
CA1978_2	MW723480	181.925	38.59
NU1979	MW723481	181.859	38.60
OR1984	MW800838	181.924	38.58
NU1986	MW723482	181.788	38.59
NU1990_1	MW723482	181.751	38.57
NU1991_2	MW723484	181.741	38.57
NU1991_3	MW723485	181.725	38.57
NU1991_7	MW723486	181.750	38.57
OR1993_1	MW723487	181.741	38.58
NU1993_2	MW723488	181.748	38.57
NU1995_2	MW723489	181.753	38.57
NU1995_3	MW723490	181.740	38.57
NU1995_4	MW723491	181.697	38.58
44076_2004	MW723500	181.755	38.57
74377_2004	MW723496	181.753	38.57
22649_2005	MW723497	181.753	38.57
72398WB_2005	MW723495	181.754	38.57
72912WB_2007	MW723498	181.804	38.57
4996WB_2008	MW723492	181.751	38.57
23221_2008	MW723494	181.770	38.57
22137_2008	MW723499	181.751	38.57
46830_2008	MW723493	181.746	38.57
31208_2011	MW736612	181.684	38.57
63525WB_2012	MW736603	181.733	38.57
2019WB_2012	MW736598	181.742	38.57
30322_2013	MW736600	181.745	38.57
32516_2013	MW736607	181.758	38.57
47039_2013	MW736597	181.738	38.57
49179WB_2013	MW736601	181.793	38.57
98039_2013	MW736599	181.753	38.57

113049_2013	MW736608	181.754	38.57
51268_2014	MW736605	181.753	38.56
6396WB_2015	MW736609	181.756	38.57
15998_2015	MW736604	181.761	38.56
28928_2015	MW736610	181.738	38.57
33747WB_2015	MW736613	181.753	38.57
53706_2016	MW736602	181.813	38.57
34403WB_2017	MW736606	181.759	38.56
54684_2018	MW647171	181.758	38.57
56140_2018	MW736611	181.759	38.56
