# Supplementary material for: A Deeper Insight into Evolutionary Patterns and Phylogenetic History of ASFV Epidemics in Sardinia (Italy) through Extensive Genomic Sequencing
Source: Viruses. 2021 Oct 4;13(10):1994. doi: 10.3390/v13101994 (PMC8539718; doi:10.3390/v13101994)
Supplement: Supplementary file 1 [file viruses-13-01994-s001.zip › S2 Table.pdf]

S2 Table. Samples coverage and diagnostic centre of the strains sequenced in this study

ID Sample	Diagnostic centre-NGS	Coverage [Mean (SD), Median]
113049_2013	A	181.42 (41.86), 179
11484WB_2014	B	25.20 (10.80), 25
1537WB_2008	B	124.99 (78.97), 114
15998_2015	A	245.74 (31.54), 250
1628WB_2009	A	6.48 (2.97), 6
2019WB_2012	A	68.96 (16.28), 69
22137_2008	A	182.81 (49.51), 178
22649_2005	A	134.31 (32.25), 133
22943_2008	A	17.30 (5.86), 17
23221_2008	A	245.24 (32.31), 250
24225_2002	B	25.61 (10.31), 25
25185_2008	A	12.52 (4.50), 12
28170_2009	B	14.92 (9.28), 14
28928_2015	A	36.84 (9.27), 37
30322_2013	A	46.45 (14.62), 45
31208_2011	B	245.97 (31.37), 250
31479_2015	B	30.23 (10.86), 30
32516_2013	A	206.32 (42.08), 209
3312_2017	B	71.69 (49.89), 62
33747WB_2015	A	62.25 (13.60), 62
34403WB_2017	A	245.42 (31.98), 250
35479_2014	A	11.70 (4.46), 11
44076_2004	A	127.62 (29.92), 126
45539_2004	B	13.55 (5.28), 13
46830_2008	A	174.07 (56.83), 169
47039_2013	A	47.09 (15.87), 45
49179WB_2013	A	66.33 (20.94), 63
4996WB_2008	A	224.23 (41.20), 248
51268_2014	A	223.92 (39.37), 237
52060_2018	B	23.90 (9.37), 24
53706_2016	A	241.57 (34.87), 250
54684_2018	A	243.52 (33.23), 250
56140_2018	A	245.31 (32.10), 250
63525WB_2012	A	43.74 (15.11), 41
6396WB_2015	A	245.70 (31.63), 250
72398WB_2005	A	178.49 (50.87), 173
72912WB_2007	A	90.06 (23.67), 89
74377_2004	A	133.37 (30.84), 132
8343_2018	B	20.68 (8.19), 20
98039_2013	A	245.21 (32.30), 250
CA1978_2	A	34.07 (9.90), 34
ITALY1983	B	93.68 (62.08), 84
NU1979	B	245.15 (32.62), 250
NU1981_2	B	85.367 (60.90), 74
NU1986	A	223.45 (40.74), 237
NU1990_1	A	212.51 (45.71), 225
NU1990_2	A	6.22 (2.98), 6
NU1991_2	A	68.66 (17.96), 68
NU1991_3	A	20.81 (6.55), 20

NU1991_7	A	95.64 (24.65), 94
NU1991_9	A	6.07 (2.87), 6
NU1993_2	A	104.41 (26.20), 103
NU1995_2	A	114.57 (27.43), 114
NU1995_3	A	35.67 (9.41), 36
NU1995_4	A	17.14 (5.52), 17
OR1984	A	221.23 (40.31), 232
OR1993_1	A	41.59 (11.58), 41
SS1981	A	10.09 (4.00), 10

A = AMES Group NAPOLI; B = CRS4 Pula
