# Supplementary figures and images for: A Deeper Insight into Evolutionary Patterns and Phylogenetic History of ASFV Epidemics in Sardinia (Italy) through Extensive Genomic Sequencing
Source: Viruses. 2021 Oct 4;13(10):1994. doi: 10.3390/v13101994 (PMC8539718; doi:10.3390/v13101994)

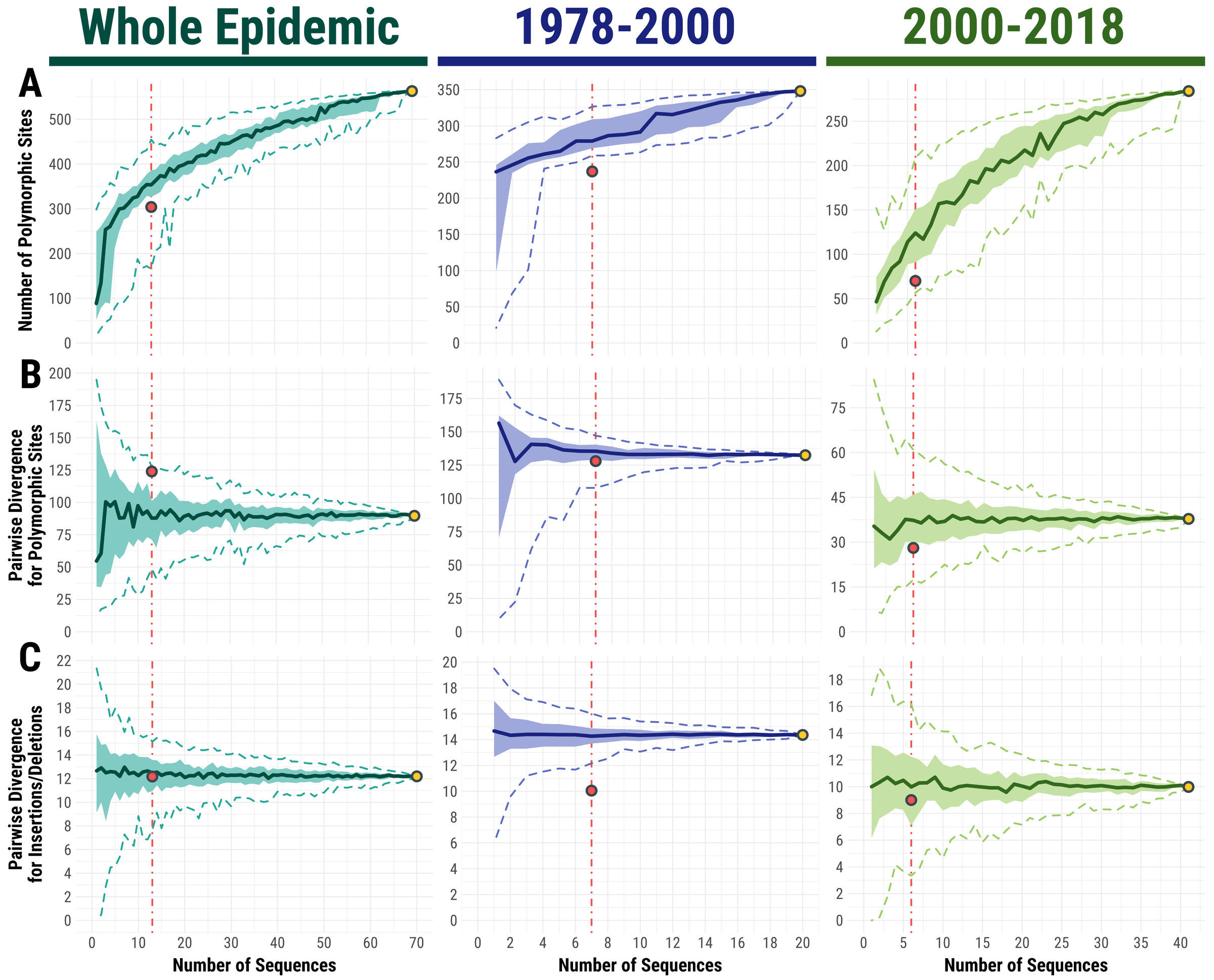

Supplement: Supplementary file 1 [file viruses-13-01994-s001.zip › Fig_S1.jpg]

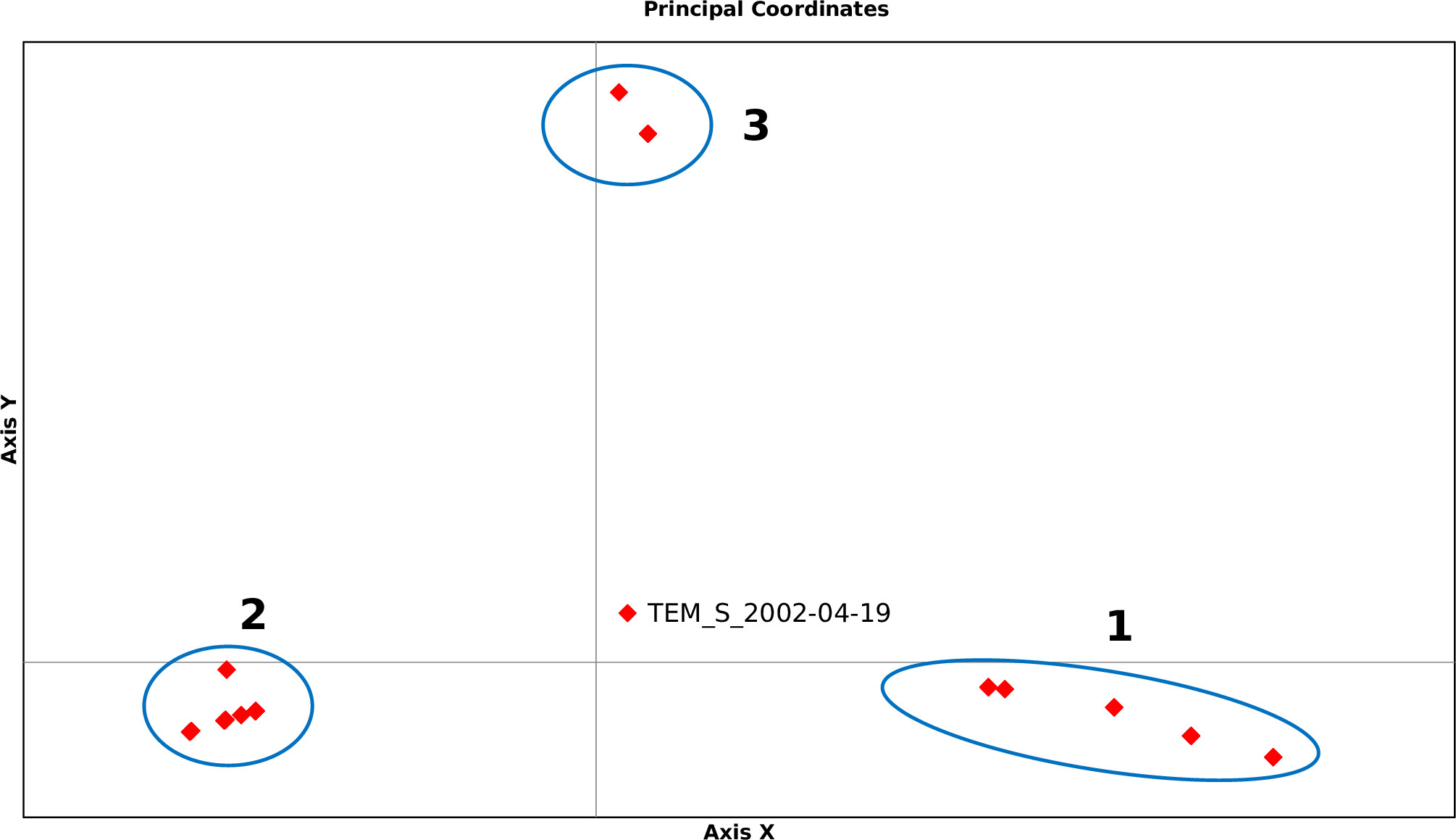

Supplement: Supplementary file 1 [file viruses-13-01994-s001.zip › Fig_S2.jpg]
